# Vertical Guided Bone Regeneration with Mineralized Cancellous Bone Allograft in a Severe Anterior Maxillary Defect: A Clinical Report with 14-Year Follow-Up

**DOI:** 10.1155/2019/6725351

**Published:** 2019-11-18

**Authors:** Gerardo La Monaca, Nicola Pranno, Giorgio Pompa, Susanna Annibali, Iole Vozza, Maria Paola Cristalli

**Affiliations:** ^1^Department of Oral and Maxillo-Facial Sciences, Sapienza University of Rome, Rome, Italy; ^2^Department of Biotechnologies and Medical Surgical Sciences, Sapienza University of Rome, Rome, Italy

## Abstract

**Purpose:**

To report the supracrestal bone regeneration of approximately 10 mm using solvent-dehydrated mineralized cancellous bone allograft and nonresorbable membrane in rehabilitation of unsuccessful implants in the anterior maxilla and stability of the regenerated bone at the 14th-year follow-up.

**Case Presentation:**

A 24-year-old female patient with a history of anterior dentoalveolar trauma resulting in the loss of upper left incisors and canine underwent rehabilitation several years ago with three implant-supported fixed prostheses. The prosthesis was both functionally and aesthetically inadequate, and the patient complained of gingival swelling, bleeding, and food impaction at the site of the implants. A staged approach for retreatment was planned, wherein the first surgical stage aimed at removing the existing implants and preparing the bone ridge and soft tissues for the augmentation procedure. The second stage was vertical ridge augmentation and simultaneous prosthetic-driven placement of two new implants at the sites of the left central incisor and canine. After nine months of uneventful healing, complete regeneration of the bony defect was achieved, and the new prosthetic rehabilitation satisfied both functional and aesthetic requirements.

**Conclusion:**

The therapeutic approach followed in the present case proved effective in achieving satisfactory functional and aesthetic results and in maintaining the stability of the regenerated bone at 14 years of follow-up.

## 1. Introduction

Clinical management of implant-supported restorations in the anterior maxillary region is complicated due to the requirement to achieve optimal aesthetic outcomes, such as suitable width and position of the keratinized soft tissue, convex buccal contour of the alveolar process, harmonious gingival margins around implants and adjacent teeth, and proper size and shape of the papillae [1, 2].

The aesthetics of the peri-implant soft tissues depends on not only the height and thickness of the underlying supporting bone and interproximal bone height of adjacent teeth but also the appropriate three-dimensional position and size of the implants [3–5].

Indeed, positioning the implant shoulder too far facially can result in a potential risk of soft tissue recession, while too far palatal placement can result in ridge-lap restorations [2, 3]. An implant positioned too close to the natural teeth causes resorption of the interproximal bone, affecting papillary support. Similarly, implants positioned too deep in the alveolar ridge can lead to increased bone loss, due to the microgap at the implant-abutment interface, which will require a long prosthetic crown, use of pink porcelain, or visible metal margins [2].

Furthermore, the extended edentulous gap and severe bone loss due to trauma present adjunctive problems, making it more questionable to achieve aesthetic results in the anterior maxillary region. In this situation, unsuccessful treatment outcomes can lead to clinical disasters, which require to be corrected with the removal of the implants and augmentation procedures to regenerate adequate volumes of bone in order to place new implants with a prosthetic-driven approach [2].

Among the different techniques proposed in the literature, vertical guided bone regeneration (v-GBR) has demonstrated predictable and effective outcomes of supracrestal bone regeneration in animal experiments and human clinical trials, allowing the correct positioning of implants [6–16].

The purpose of this case report is to report vertical bone regeneration of approximately 10 mm using solvent-dehydrated mineralized cancellous bone allograft and nonresorbable membrane in the rehabilitation of unsuccessful implants in the anterior maxilla and stability of the regenerated bone at 14 years of follow-up.

## 2. Clinical Report

**Figure 1:**
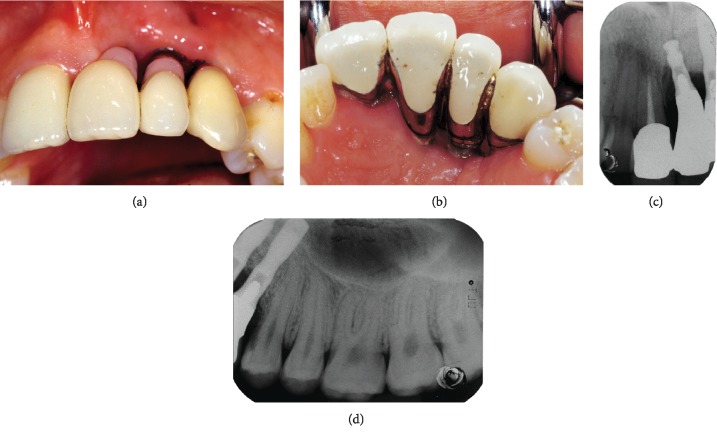
Preoperative clinical and radiographic views: (a) buccal aspect, (b) palatal aspect, (c) periapical radiograph of the right central incisor, (d) periapical radiograph of the left first premolar.

A 24-year-old healthy female patient with a history of anterior dentoalveolar trauma resulting in loss of the upper left incisors and canine underwent rehabilitation several years ago with three implant-supported fixed prostheses. The prosthesis was both functionally and aesthetically inadequate, and the patient complained of gingival swelling, bleeding, and food impaction at the site of the implants (Figures 1(a) and 1(b)). Standard periapical dental radiographs taken to assess periodontal status of the right central incisor (Figure 1(c)) and left first premolar (Figure 1(d)) adjacent to the implants showed significant bone loss on the mesial aspect of the roots.

**Figure 2:**
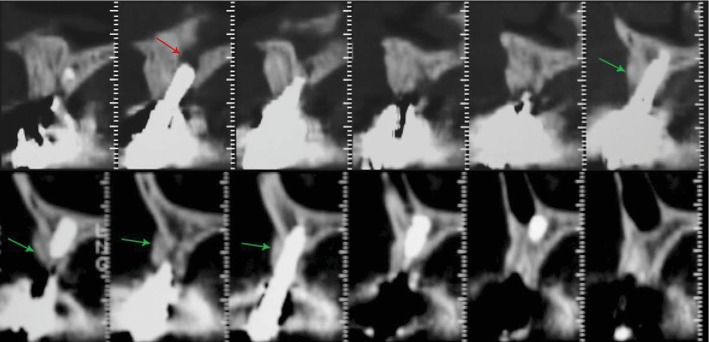
Computed tomography scans showed the implant in the region of the central incisor inside the incisive canal (red arrow) and the three-dimensional bone loss around the implants, which were osseointegrated only in the apical portion with the remaining surfaces being covered by radiopaque biomaterial particles encapsulated in the soft tissues (green arrows).

Computed tomography scans showed three-dimensional bone loss around implants, which were osseointegrated only in the apical portion. The remaining surfaces of the implants were covered by radiopaque biomaterial particles encapsulated in the soft tissue, and the implant in the region of the central incisor was inside the incisive canal (Figure 2).

Treatment planning involved removal of pre-existing prosthesis and implants, reconstruction of the bony ridge by a v-GBR procedure, and simultaneous insertion of two new implants with a prosthetic driven approach. As vertical ridge augmentation is affected by the interproximal height of the bone adjacent to the defect, the roots of the right central incisor and left first premolar were endodontically treated in order to be submerged under the membrane after decoronation, for utilizing the regenerative potential of their distal interproximal bone.

For all interventions, the patient received preoperative antibiotic and analgesic therapy with amoxicillin 875 mg+clavulanic acid 125 mg (Augmentin, GlaxoSmithKline S.P.A., Verona, Italy) and ibuprofen 600 mg (Brufen, Abbott, Rome, Italy) 1 hour prior to surgery and were repeated twice daily postoperatively.

Surgical procedures were performed after oral sedation with diazepam 0.25 mg/kg (Valium gtt 25 ml/5%, Roche S.P.A., Monza, Italy), administered 30 minutes preoperatively, under local anaesthesia with mepivacaine 2% with adrenalin 1 : 100,000 (Carbocaine, AstraZeneca, Italy).

**Figure 3:**
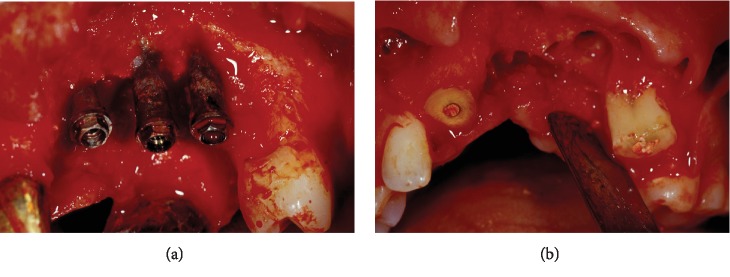
The first intervention: (a) clinical view of the implants and grafting material after flap elevation and (b) the bone ridge after removing implants and cutting the crowns of the adjacent teeth.

The first surgical stage was aimed at removing the implants and preparing the bone ridge and soft tissues for augmentation procedure (Figures 3(a) and 3(b)). A full-thickness flap was raised, and the implants, grafting material (hydroxyapatite), and granulation tissue were removed. The crowns of the adjacent teeth were cut, and the roots, after scaling and surface debridement, were submerged under the flap, which was coronally repositioned by means of periosteal releasing incisions. To support the aesthetic needs of the patient, a removable partial denture, with metal subframework and rests on posterior teeth, was provided to avoid transmucosal pressure on the surgical area.

**Figure 4:**
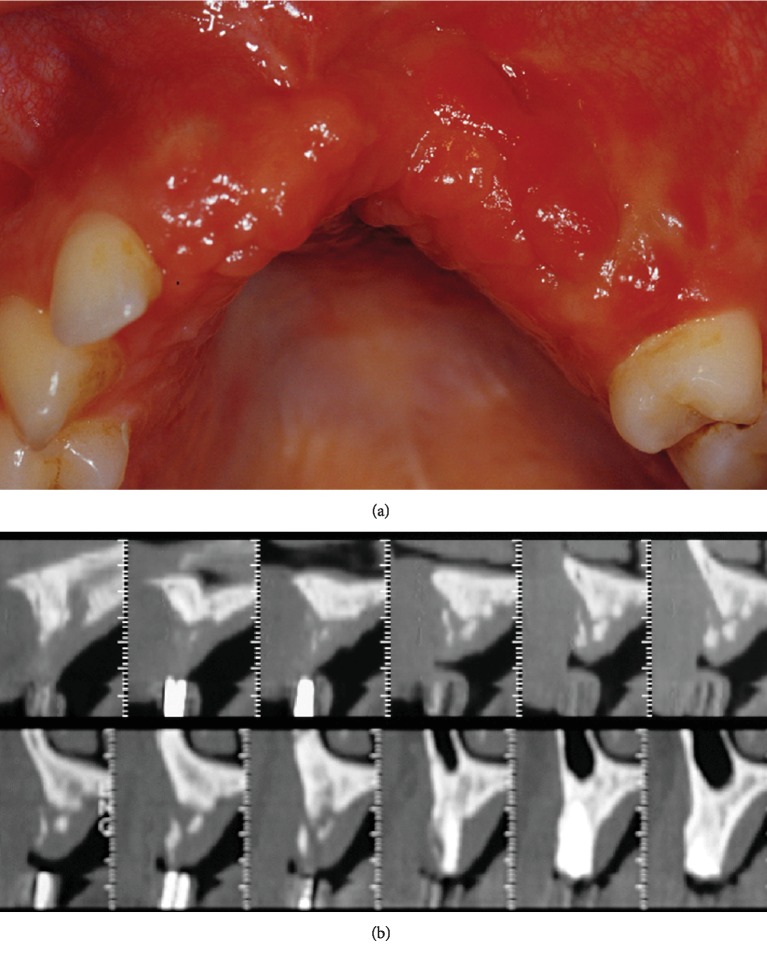
After two months of uneventful healing: (a) clinical view and (b) computed tomography scans showing the three-dimensional ridge defect and height of the available bone (5 mm) in the implant sites indicated by the template radiopaque markers.

After two months of uneventful healing (Figure 4(a)), wax-up of the prosthetic restoration was made on diagnostic/study mounted casts in order to identify the ideal three-dimensional position of the implants. A template was then fabricated to be used in the radiographic diagnosis of the selected implant sites and during surgery as a guide for correct insertion of the implants.

Postoperative computed tomography scans (Figure 4(b)) showed residual bone height of 5 mm and a three-dimensional bone defect of approximately 10 mm at the planned implant sites, corresponding to the radiopaque markers on the template. Spread of some hydroxyapatite granules into the soft tissues was also observed.

Based on clinical and radiographic information, the second intervention was planned for vertical ridge augmentation and simultaneous prosthetic driven placement of two implants at the sites of the left central incisor and canine.

**Figure 5:**
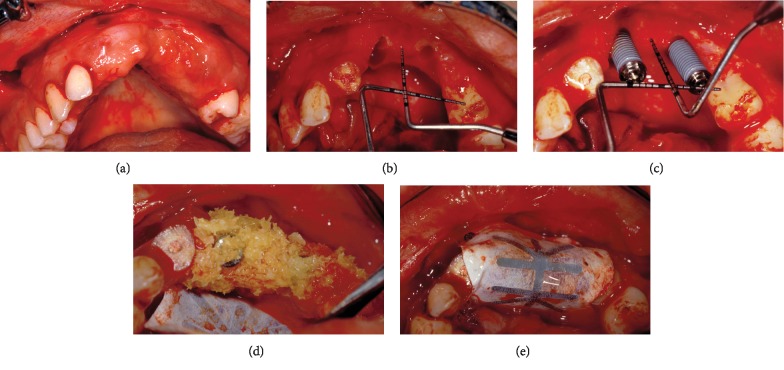
The second intervention: (a) the full-thickness trapezoidal flap, (b) buccal view of the vertical defect in the anterior maxilla, (c) the implants protruding 10 mm from the top of the crest of the bone; (d) particulate composite bone graft in place and e-PTFE membrane fixed on the palate, and (e) e-PTFE membrane fixed over the grafted area with buccal titanium miniscrews.

A full-thickness trapezoidal flap (Figure 5(a)) with wide buccal base and palatal pedicle was performed in order to ensure good vascular supply to the grafted area and tension-free complete closure over the augmented ridge. The horizontal incision was performed within the mucosa of the buccal fold and was connected with two divergent vertical incisions, distal to the right canine and left second premolar. The incisions were extended intrasulcularly all around the teeth mesial to the defect. The flap was elevated with great caution to avoid perforations due to the presence of scar tissue from the previous surgery followed by the removal of fibrous tissue. The bony defect, as measured with two crossed-calibrated periodontal probes, extended 10 mm from its most apical portion to a line connecting the cementoenamel junction of the adjacent teeth (Figure 5(b)). Two implants (MKIV 4 × 15 mm, Branemark System®, Nobel Biocare), protruding for about 10 mm from the top of the bony crest, were placed in the three dimensionally corrected position using the radiographic/surgical template (Figure 5(c)). A nonresorbable titanium-reinforced e-PTFE membrane (W. L. Gore & Associates, Flagstaff, AZ, USA) was trimmed, adapted to the defect, and fixed on the palatal plate using two titanium miniscrews. Multiple perforations of the cortical bone were made with a small round bur in order to open the medullary spaces and increase bleeding. Following this, exposed implant threads were covered with autogenous bone chips that were harvested using a bone scraper (Safescraper; META, Reggio Emilia, Italy) from the mandibular ramus. The autograft was enhanced with a second layer of solvent-dehydrated mineralized cancellous bone allograft (Puros® allograft, Zimmer Dental) (Figure 5(d)). The membrane was stabilized over the augmented area with two additional buccal titanium self-tapping screws (Figure 5(e)). The flap was then coronally advanced by means of periosteal releasing incisions in the buccal fold and sutured with two lines of closure (GORE-TEX CV-5 Suture, W.L. Gore & Associates), to be removed on the fourteenth day. Horizontal mattress sutures were given on the horizontal incision facing at least 3 mm of the marginal connective tissue, in order to prevent exposure of the membrane. Interrupted sutures were given at a more coronal position on the horizontal and vertical incisions to seal the wound.

**Figure 6:**
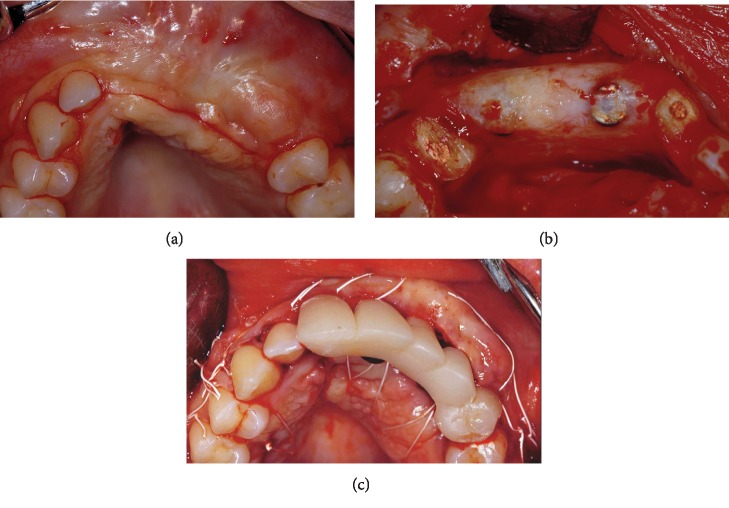
At the 9th month re-entry: (a) clinical view of the regenerated area, (b) alveolar crest regeneration after flap elevation and membrane removal, and (c) flap sutured after free connective tissue graft and first temporary bridges.

At the surgical re-entry after 9 months of uneventful healing (Figure 6(a)), the miniscrews and titanium-reinforced e-PTFE membrane were removed. Complete filling of the bony defect was observed with a hard regenerated tissue clinically similar to bone, covered by a thin layer of firm connective tissue (Figure 6(b)). At the same time, healing abutments were connected to the implants, the right central incisor and left first premolar roots were prepared to receive gold-cast post and cores to support an interim bridge. The flap was apically repositioned to restore the vestibular depth lost during the second surgical stage, and a free connective tissue graft, harvested from the palatal mucosa, was performed to improve the aesthetics of the soft tissues (Figure 6(c)). After 2 weeks, the gold-cast post and cores were cemented inside the roots. A second temporary bridge with two single crowns supported by the teeth was fixed along with a three-unit bridge supported by two implants, to guide morphological maturation of the soft tissue and stamp the ovate pontic.

**Figure 7:**
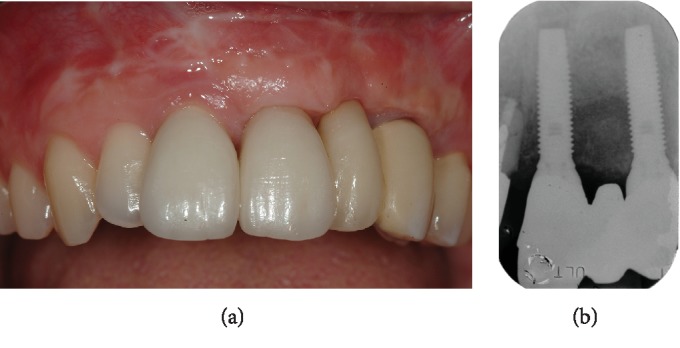
Definitive cemented prosthetic restoration in porcelain-fused-to-metal: (a) clinical view and (b) periapical radiograph.

The definitive cemented prosthetic restoration in porcelain-fused-to-metal was fixed after 6 months with two single crowns supported by the teeth and a three-unit implant-supported bridge (Figures 7(a) and 7(b)).

**Figure 8:**
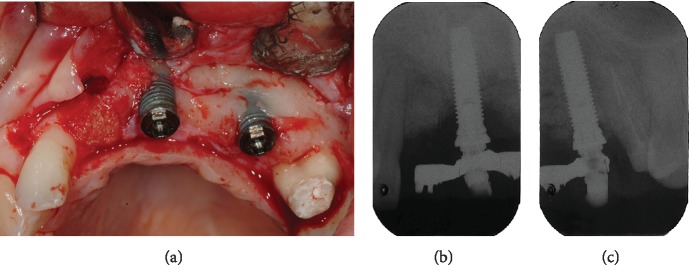
The regenerated bone after 14 years of loading, during surgery to treat the peri-implantitis lesions on the buccal surface of the implants and after extraction of the right central incisor: (a) clinical view, (b) periapical radiograph of mesial implant, and (c) periapical radiograph of distal implant.

After 14 years of loading, the stability and quality of the regenerated bone was clinically and radiographically assessed during surgery to remove the right central incisor and treat peri-implantitis on the buccal surface of the implants (Figure 8).

## 3. Discussion

In the presented case, failure of the pre-existing implants in the anterior maxilla led to a complex clinical situation, which necessitated the removal of the implants. Additional bone and soft tissue augmentation procedures and new implant-prosthetic treatment were also indicated in order to achieve satisfactory aesthetics, function, and long-term stability of the reconstructed peri-implant tissues.

Keeping in mind the challenging site, the anterior maxillary aesthetic zone, anatomic and pathologic factors, such as the pre-existing unsuccessful implant rehabilitation and residual three-dimensional bone defect of approximately 10 mm, and surgical experience and skill of the surgeon, the decision-making process for the treatment planning was based on scientific evidence [2].

Among the different procedures proposed for vertical ridge augmentation (distraction osteogenesis, block graft, and guided bone regeneration), v-GBR with cancellous bone allograft and titanium-reinforced e-PTFE membrane was chosen for treatment in this clinical case [17, 18].

Clinical and histological data reported in the literature in the last decade appear to support the v-GBR procedure as an effective treatment to augment the lost bony structure and thus allow positioning of the implants [9–13].

In a review on augmentation procedures for rehabilitation of deficient edentulous ridges with oral implants, Chiapasco and coworkers reported bone gain of 2-7 mm following v-GBR. The study also reported an overall survival rate of 92% to 100% in implants placed in the augmented sites with nonresorbable barriers, while success rates ranged from 61.5% to 100% [9].

In another systematic review, Aghaloo and Moy reported more detailed documentation and long-term follow-up studies on GBR procedures than other alveolar ridge augmentation techniques. However, they reported that implant survival rates were not significantly different from those in nonaugmentated sites [10].

The results of the systematic review by Rocchietta and coworkers were consistent with those previously reported by Chiapasco and coworkers. The review reported vertical bone gain of 2-8 mm, implant survival rates of 92.1% to 100% over 1-7 years of follow-up, and success rates ranging from 61.5% to 97.5% [11].

In 2014, a systematic review by Milinkovic and Cordaro aimed at establishing the clinical indications for various bone augmentation procedures based on type and dimensions of the defect. The review reported an average linear bone gain of 3.04 mm, mean implant survival rate of 98.9%, and mean complication rate of 13.1% for vertical defects treated with simultaneous GBR [13].

In a more recent systematic review and meta-analysis, Urban and coworkers [12] reported the following data:

- The weighted mean clinical vertical bone gain of 4.18 mm after GBR was lower than that after distraction osteogenesis (8.4 mm) and greater when compared to bone blocks (3.46 mm). The main findings of the meta-analysis, based on the changes in baseline and final values, showed that in v-GBR technique, clinical bone gain was influenced by the type of particulate grafting material and nonresorbable membrane
- The incidence of complications following GBR (12.1%) was lower than that observed after distraction osteogenesis and bone blocks (47.3% and 23.9%, respectively). Furthermore, in line with previous systematic reviews, resorbable membranes were more prone to complications than nonresorbable membranes (22.7% versus 6.9%)
- The implant success rates ranging between 85.33% and 100% and the aggregated mean implant survival rate (98.95%, range: 90.5-100%) were comparable to previous studies that reported on implants placed in pristine sites
- To obtain adequate vertical bone gain in large defects, e-PTFE membranes have proven effective in preventing migration of epithelial cells and fibroblasts, in maintaining space, and in stabilizing graft materials [19]

Although autogenous bone is considered the “gold standard,” many bone substitutes have been used in augmentation procedures to decrease the extent of surgical invasion and postoperative discomfort. The efficacy of the bone allograft used in the present case in promoting bone formation has been reported in many studies and in different augmentation procedures [6, 7, 20–26]. Indeed, the processing of solvent-dehydrated mineralized cancellous bone, which preserves the natural collagen matrix, trabecular pattern, and porosity of human cancellous bone, would have better osteoconductive potential compared to freeze-dried bone allografts [19, 22]. Furthermore, the presence of calcium and phosphate would result in less reabsorption than demineralized bone allografts, thus maintaining the space for a longer period [23].

The rationale behind the combination of bone allograft and autologous bone chips was to associate the osteoconductive properties of mineralized cancellous allograft with osteogenic and osteoinductive properties of the autogenous bone. Bone allograft acts as a scaffold for space creation and maintenance and autologous bone initiates the regenerative process, releasing osteoblasts and growth factors [27]. Furthermore, the use of a bone scraper for harvesting bone chips minimizes donor site morbidity and shortens the collection time.

The two-stage approach, with removal of the existing prosthesis and implants prior to the augmentation procedure, was necessary to eradicate the infection and enhance the quality and quantity of soft tissues at the recipient site. In addition, tension-free complete soft tissue closure over the augmented area could be obtained, which is crucial for success of the procedure [28]. Furthermore, although studies have reported more bone gain with delayed implant placement after the GBR procedure, in the present case, simultaneous placement of implants insertion was chosen to avoid an additional surgical intervention.

Lastly, despite the increased risk of peri-implantitis in augmented sites compared with pristine sites, the peri-implantitis lesion in this case developed at the 14th year of follow-up [29]. This may be attributed to low compliance with the maintenance protocol and not to the augmentation technique or graft material used [28].

## 4. Conclusions

In the present case, the therapeutic approach to the severe anterior maxillary defect, based on v-GBR and mineralized cancellous bone allograft, resulted in satisfactory aesthetic and functional prosthetic rehabilitation. In addition, the clinical assessment at 14 years of follow-up showed the three-dimensional stability of the regenerated bone, despite the loss of the adjacent right central incisor and the onset of peri-implantitis.

The positive outcomes were closely related to the awareness of ridge anatomy, soft and hard tissue deficiencies, preoperative prosthetic planning, three-dimensional correct positioning of implants with a prosthetic driven approach, strict v-GBR surgical protocol, careful handling of soft tissues, and appropriate provisional and definitive prosthetic rehabilitation.

No ethical committee approval was sought to start this clinical report study since this was not required by national legislation or any ordinance of the local inspection authority.
